# Temporal stability of bacterial symbionts in a temperate ascidian

**DOI:** 10.3389/fmicb.2015.01022

**Published:** 2015-09-24

**Authors:** Susanna López-Legentil, Xavier Turon, Roger Espluga, Patrick M. Erwin

**Affiliations:** ^1^Department of Biology & Marine Biology and Center for Marine Science, University of North Carolina WilmingtonWilmington, NC, USA; ^2^Center for Advanced Studies of Blanes – Consejo Superior de Investigaciones CientíficasBlanes, Spain; ^3^Department of Animal Biology, University of BarcelonaBarcelona, Spain

**Keywords:** Tunicata, symbiosis, Mediterranean Sea, bacteria, temporal, *Didemnum*, T-RFLP, 16S rRNA

## Abstract

In temperate seas, both bacterioplankton communities and invertebrate lifecycles follow a seasonal pattern. To investigate whether the bacterial community associated with the Mediterranean ascidian *Didemnum fulgens* exhibited similar variations, we monitored its bacterial community structure monthly for over a year using terminal restriction fragment length polymorphism and clone library analyses based on a nearly full length fragment of the 16S rRNA gene. *D. fulgens* harbored a bacterial consortium typical of ascidians, including numerous members of the phylum Proteobacteria, and a few members of the phyla Cyanobacteria and Acidobacteria. The overall bacterial community in *D. fulgens* had a distinct signature from the surrounding seawater and was stable over time and across seasonal fluctuations in temperature. Bacterial symbionts were also observed around animal cells in the tunic of adult individuals and in the inner tunic of *D. fulgens* larvae by transmission electron microscopy. Our results suggest that, as seen for sponges and corals, some species of ascidians host stable and unique bacterial communities that are at least partially inherited by their progeny by vertical transmission.

## Introduction

Ascidians, or sea-squirts, are filter-feeding organisms with an abundance and diversity of species and functions that render them critical to healthy ecosystem functioning (Lambert, 2005a,b; Shenkar and Swalla, 2011). However, ascidians are often better known as important fouling organisms, sometimes becoming invasive and disrupting the organization of native benthic communities (Lambert, 2007; Bryon and Scavia, 2008; York et al., 2008). Besides their ecological impact, ascidians also have substantial importance for biotechnology and drug discovery through the production of unique and structurally diverse secondary metabolites (Anderson et al., 2010; Paul et al., 2011; Blunt et al., 2014, 2015). In fact, although ascidians hold great potential for new drug discovery (Anderson et al., 2010), this group has received relatively less attention compared with other benthic invertebrates such as sponges and mollusks.

Ascidians have been reported to form symbiotic associations with a wide range of bacterial phyla (Martínez-García et al., 2007; Erwin et al., 2013, 2014; Tianero et al., 2015), and in particular with Proteobacteria and Bacteroidetes (Schuett et al., 2005; Martínez-García et al., 2007; Tait et al., 2007; Erwin et al., 2013, 2014; Tianero et al., 2015). To date, the best-studied symbiotic relationships among bacteria and ascidians are between the ascidian family Didemnidae and the bacterial phylum Cyanobacteria (Yokobori et al., 2006; Münchhoff et al., 2007; López-Legentil et al., 2011; Behrendt et al., 2012; Erwin et al., 2014; Tianero et al., 2015). These interactions have received quite a bit of attention since the discovery of *Prochloron*, an ascidian-associated cyanobacterium that contains a photosynthetic pigment profile more similar to green algae and plants (both chlorophyll *a* and *b*) than cyanobacteria (Lewin, 1977, 1978; Hirose et al., 2004, 2007; Oka and Hirose, 2008; Hirose and Nozawa, 2010). Other cyanobacteria species have been reported in association with ascidians, including *Synechocystis* (Lafargue and Duclaux, 1979; Hernández-Mariné et al., 1990; Shimada et al., 2003) and *Acaryochloris*, the latter of which uses chlorophyll *d* as its major photosynthetic pigment (Miyashita et al., 1996, 2003; López-Legentil et al., 2011; Martínez-García et al., 2011). Associations between ascidians and Cyanobacteria are thought to be evolutionarily ancient, widely distributed and host-specific (Hirose et al., 1996, 2005; Hirose, 2000, 2013; Hirose and Fukuda, 2006; Kojima and Hirose, 2010, 2012; López-Legentil et al., 2011). Much less is known about the symbiotic association between ascidians and bacteria other than Cyanobacteria. Evidence to date points toward a high degree of host and species-specificity (Erwin et al., 2014; Tianero et al., 2015), but much remains to be done to assess their full diversity, temporal stability, and transmission mode.

In temperate seas like the Mediterranean, the lifecycles of ascidians follow a seasonal pattern (Turon and Becerro, 1992; López-Legentil et al., 2005a,b, 2013). Temperature has often been pinpointed as the main factor triggering reproduction and growth in these animals (Millar, 1971; López-Legentil et al., 2005b, 2013), but other parameters such as resource availability, turbidity and wave exposure may also play significant roles (Millar, 1971; Ribes et al., 1998; Valentine et al., 2007; Shenkar and Loya, 2008; Ritzmann et al., 2009). In addition, reproduction appeared to be a main energy sink and was reported to significantly influence the temporal dynamics of other biological cycles in ascidians, such as growth and the production of secondary metabolites (López-Legentil et al., 2005b, 2007, 2013). In fact, the production of some secondary metabolites in temperate ascidians has also been shown to follow a seasonal pattern (López-Legentil et al., 2006, 2007). Since at least some secondary metabolites isolated from ascidians are known to be totally or partially produced by symbiotic bacteria (e.g., Aassila et al., 2003; Schmidt et al., 2005; Riesenfeld et al., 2008; Schmidt and Donia, 2010), temporal fluctuations in symbiont communities may also be predicted to exhibit seasonal patterns.

The colonial ascidian *Didemnum fulgens* (Milne-Edwards, 1841) is commonly found in the western Mediterranean Sea (Lafargue and Wahl, 1987; Koukouras et al., 1995) and observed either as an epiphyte on the rhizomes of the seagrass *Posidonia oceanica* (Balata et al., 2007) or attached to rocky substrates in the infralittoral (López-Legentil et al., 2013). *D. fulgens* broods its larvae and exhibits a seasonal life cycle characterized by alternating periods of growth and reproduction (López-Legentil et al., 2013). This species also lacks macroscopic epibionts, is a good competitor for space, and only the flatworm *Thysanozoon brocchii* has occasionally been observed grazing on it (Velasco, 2012). Clean colony surfaces and a lack of generalist predators are good indicators that colonial ascidians, such as *D. fulgens*, are actively producing (themselves or their microbial symbionts) bioactive secondary metabolites for their defense. The goal of this study was to investigate whether the bacterial community associated with the Mediterranean ascidian *D. fulgens* was subjected to seasonal variations. To address this issue, we monitored *D. fulgens* bacterial diversity monthly for over a year using terminal restriction fragment length polymorphism (T-RFLP) of bacterial 16S rRNA gene sequences. Dominant bacterial symbionts were identified by constructing clone libraries based on a fragment of the 16S rRNA gene and performing phylogenetic analyses. Symbionts were also visualized in both adults and larvae by transmission electron microscopy (TEM). To the best of our knowledge, this is the first study investigating temporal stability of bacterial symbiont communities in ascidians, and the findings reported here should provide much needed data on the intrinsic characteristics of ascidian-bacteria symbioses.

## Materials and Methods

### Sample Collection

Samples were collected at L’Escala, Spain (‘La Depuradora’: 42° 7′ 29″ N, 3° 7′ 57″ E; NW Mediterranean Sea) in August 2010 and monthly from February 2011 to May 2012 by SCUBA diving, with the exception of February 2012 due to unfavorable diving conditions (Supplementary Table S1). Sampled colonies were separated by at least 5 m and collection depth ranged from 4 to 10 m. After collection, specimens were immediately fixed in absolute ethanol and stored at -20°C until analyzed. Seawater temperature was recorded as described in López-Legentil et al. (2013) and ranged from 12°C (March 2012) to 22°C (September 2011).

### DNA Extraction and T-RFLP Analyses

A piece of tunic (<2 mm^3^) per colony was carefully dissected under a stereomicroscope to remove the zooids and any debris attached. DNA was extracted using the Animal Tissue Protocol, DNeasy^®^ Blood and Tissue kit (Qiagen^®^) and used as template for PCR amplification of a fragment of the bacterial 16S rRNA gene using the forward primer 8F (Turner et al., 1999) labeled with a 5′-end 6-carboxyfluorescein (6-FAM) and the reverse primer 1509R (Martinez-Murcia et al., 1995). Total PCR reaction volume was 25 μL, including 5 pmol of each primer, 5 nmol of each dNTP, 1x reaction buffer (Ecogen), and 2.5 units of BIOTAQ polymerase (Ecogen). The thermocycler program consisted of an initial denaturing step at 94°C for 2 min, 30–35 amplification cycles (denaturing at 94°C for 1 min, annealing at 50–55°C for 30 s and extension at 72°C for 90 s), and a final extension at 72°C for 6 min, performed on a PCR System 9700 (Applied Biosystems). The number of amplification cycles and annealing temperature were optimized for each sample (within the ranges above) to amplify sufficient product yields for downstream analyses. PCR products were gel-purified and cleaned using the QIAquick Gel Extraction kit (Qiagen^®^) and DNA concentration was measured using a Qubit^TM^ flurometer and Quant-iT^TM^ dsDNA Assay kit (Invitrogen^TM^), following manufacturers’ instructions. Approximately 40–100 ng of purified PCR products were digested separately with the restriction endonucleases *HaeIII* and *MspI* (Promega) overnight at 37°C. Following digestion, samples were ethanol precipitated and resuspended with 10 μl Hi-Di formamide and 0.5 μl of GeneScan 600-LIZ size standard (Applied Biosystems). Fragments were loaded on an automated sequencer ABI 3730 (Applied Biosystems) available at the Genomics Unit of the Scientific and Technologic Center of the University of Barcelona (Spain) and analyzed using Peak Scanner v. 1.0 (Applied Biosystems). Fragment lengths in the range of 50–600 bp were considered for further analysis and imported into the program T-REX (Culman et al., 2009). For noise reduction prior to terminal restriction fragment (T-RF) alignment, the filtering algorithm of Abdo et al. (2006) was used to eliminate background noise (standard deviation multiplier = 3) and samples with low total fluorescence. T-RFs were then aligned across samples using a 1-bp clustering threshold and peak profiles were standardized using relative abundance (percentage total fluorescence).

### Comparison with Seawater Bacterial Communities

To compare free-living, seawater bacterial composition with bacterial profiles from *D. fulgens*, bacterioplankton data from a previous study (Erwin et al., 2012a) were used. Due to logistical constraints, seawater samples were unable to be collected with ascidian samples for this study; however, these data from the previous study were (1) collected during the same time period (September and December 2010, and March and June 2011), (2) collected at two nearby sites (<35 km apart; Tossa de Mar 41°43′ 13.62″ N, 2°56′ 26.90″ E; and Blanes 41°40′ 54.87″ N, 2°49′ 0.01″ E) located a similar distance offshore (<100 m) from the same depth range (5–10 m) over comparable benthic habitat, and (3) processed using the same PCR primer pair and T-RFLP processing pipeline utilized herein. Further, our previous work has shown similar surface bacterioplankton communities across 100s of km in the NW Mediterranean Sea (Pita et al., 2013). Full details of sample collection and processing are found in Erwin et al. (2012a); in short, triplicate samples seawater (500 ml each) were concentrated on 0.2 μm filters, stored at -80°C and extracted using the same DNA extraction kit (DNeasy^®^ Blood and Tissue kit) and protocol (Animal Tissue) utilized herein for ascidian samples.

### Statistical Analyses

To compare bacterial community structure among samples, we constructed Bray–Curtis similarity matrices using square root transformations of relative T-RF abundance data and visualized the results in non-metric multi-dimensional scaling (nMDS) plots. To determine whether the bacterial community in *D. fulgens* was stable over time, similarity matrices were analyzed with nested permutational multivariate analysis of variance (PERMANOVA) with the factors season and month (within season). In addition, PERMANOVA with the single factor month was performed to ensure that non-seasonal temporal patterns were not missed by forcing monthly data into ‘seasonal’ categories. PERMANOVA pairwise comparisons were corrected based on the Benjamini-Yekutieli (B-Y) false discovery rate control (Benjamini and Yekutieli, 2001) and an experiment-wise error rate of 0.05. Finally, permutational multivariate analyses of dispersion (PERMDISP) were conducted to test for heterogeneity of dispersion among seasons and months. PERMANOVA and PERMDISP analyses were conducted using PERMANOVA+ implemented in Primer v. 6 (Plymouth Marine Laboratory, UK).

To determine the putative identity of T-RFLP profile peaks, *in silico* digestions of 16S rRNA gene sequences (see below) were performed in Geneious v. 8 (Kearse et al., 2012). Cut sites at the 5′-end of each sequence were identified based on the recognition sequence of the restriction endonucleases *HaeIII* (GG’CC) and *MspI* (C’CGG) and utilized to predict the corresponding length of sequences in T-RFLP profiles. In addition to T-RF peak identification, comparing clone libraries and T-RFLP profiles help to determine the specificity of individual T-RFs. Identical or closely related sequences that match to a single T-RF provide evidence for a phylotype-specific peak, while unrelated sequences that match to a same T-RF indicate a multiple phylotype peak.

### 16S rRNA Clone Libraries and Phylogenetic Analyses

Clone libraries based on a fragment of the 16S rRNA gene sequence were constructed for two colonies of *D. fulgens,* collected in April 2011 (DF2) and May 2011 (DF7), to recover near full-length 16S rRNA gene sequences. Clone libraries were built with the same primer pair used for T-RFLP analysis (without the 6-FAM label) as described previously (Erwin et al., 2012a). All clones obtained were sent for purification and sequencing to Macrogen, Inc. (Seoul, Korea). Raw sequence reads were processed in Geneious v. 8 (Kearse et al., 2012) by aligning forward and reverse reads to yield a final consensus sequence for each clone. Quality-checked sequences are archived in GenBank under accession numbers KR348488-KR348508.

Bacterial sequences were ascribed to operational taxonomic units (OTUs) based on 99% sequence identity (nearest-neighbor algorithm). Representative sequences from each 99% OTU were analyzed using the Ribosomal Database Project II (Cole et al., 2007) sequence classifier and the BLASTn tool from GenBank to assess taxonomic affiliations and check for sequencing artifacts (e.g., chimeras). Sampling coverage of clone libraries was calculated using the bootstrap estimator (Smith and van Belle, 1984). For phylogenetic analyses, all recovered sequences herein and reference sequences from GenBank were aligned using Clustal W v. 2 (Larkin et al., 2007) with a gap opening penalty of 24 and a gap extension penalty of 4, values appropriate for aligning gene sequences (e.g., 16S rRNA) with multiple variable and conserved regions (Erwin and Thacker, 2007). To build phylogenetic trees, neighbor-joining (NJ) and maximum likelihood (ML) analyses were conducted in MEGA v. 5.2.2 (Tamura et al., 2007). For NJ analyses, the Jukes-Cantor model of nucleotide substitution was used and data were re-sampled using 1,000 bootstrap replicates (Felsenstein, 1985). The ML tree was built based on the GTR+I+G (Tavaré, 1986) model with substitution rates varying among sites according to an invariant and gamma distribution and 100 bootstrap replicates.

### Transmission Electron Microscopy Analyses

The ultrastructure of the most commonly occurring bacteria in the tunic and the larva of *D. fulgens* were examined by TEM. In May 2011, a mature colony was transported alive to the laboratory and a small piece of tunic (ca. 2 mm^3^) and a larva were carefully isolated under a stereomicroscope and immediately were fixed in 2.5% glutaraldehyde and 2% paraformaldehyde using filtered seawater as buffer. Samples were incubated in the fixative mixture overnight at 4°C, washed several times in filtered seawater and stored at 4°C until processed. To construct resin blocks, samples were dehydrated in a graded ethanol series and embedded in Spurr’s resin at room temperature. Semi-thin (five microns) and ultrathin sections (ca. 60 nm) were cut with a Reichert Ultracut microtome. Ultrathin sections were stained with uranyl acetate and lead citrate for ultrastructural observation (Reynolds, 1963). TEM observations were conducted on a JEOL JEM-1010 (Tokyo, Japan) electron microscope coupled with a Bioscan 972 camera (Gatan, Germany). Resin blocks, ultrathin sections and TEM observations were performed at the Microscopy Unit of the Scientific and Technologic Center of the University of Barcelona.

## Results

### Bacterial Community Structure over Time and Specificity

**FIGURE 1:**
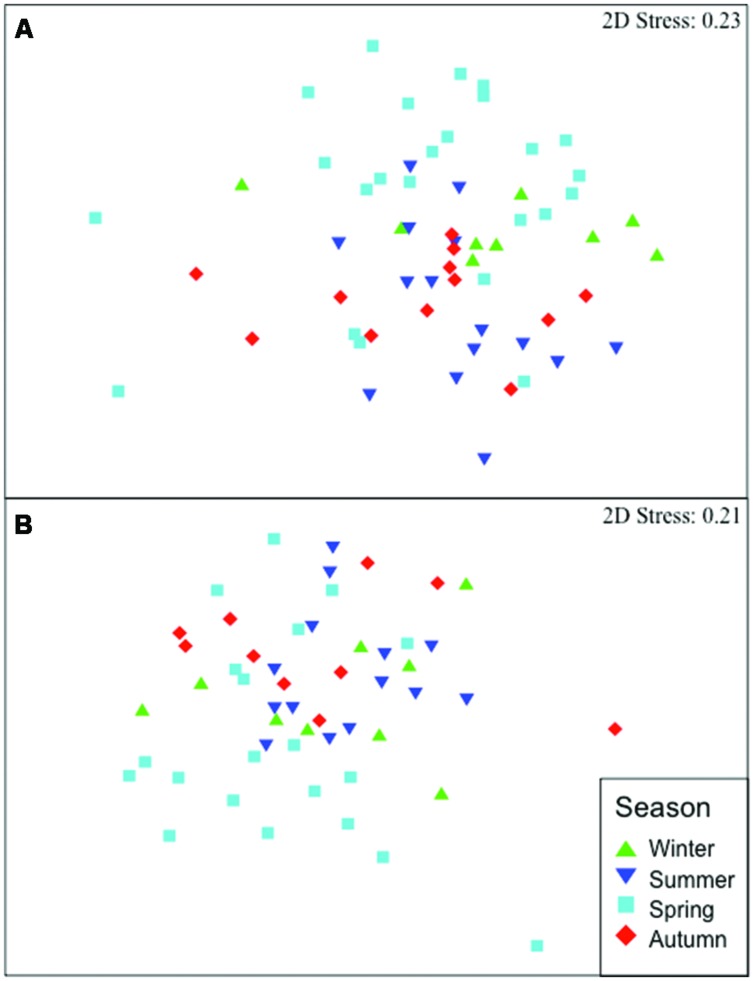
**Non-metric multi-dimensional scaling (nMDS) plots of bacterial community similarity in *Didemnum fulgens* over the 16 months of study and for each season.** nMDS ordination based on Bray-Curtis similarity of T-RFLP profiles for *HaeIII* **(A)** and *MspI* **(B)** datasets. Stress values (goodness-of-fit between two-dimensional ordination distances and similarity matrix distances) are shown in parenthesis for each enzyme (range = 0 to 1, lower values = better fit).

Symbiont communities within *D. fulgens* exhibited stability throughout the monitoring period, averaging 62 and 62.2% community similarity in T-RFLP profiles (*HaeIII* and *MspI*, respectively) across all samples. nMDS plots showed a lack of seasonal structure, with no consistent clustering of bacterial communities by season or month for either restriction enzyme dataset (**Figure 1** and Supplementary Figure S1 respectively). While statistical analyses of community structure (PERMANOVA) revealed significant variability in structure across all seasons (*p* < 0.023, **Table 1**), no significant pairwise comparisons were detected between seasons. Similarly, significant variability in structure were detected across all months (PERMANOVA, *p* = 0.001 in both nested and single factor analyses; **Table 1**), yet no pairwise comparisons were significant between months nested within seasons. Further, full pairwise comparisons between all months (single factor analysis) revealed that after B-Y correction, only three pairwise comparisons (of 120 total) were significant for *HaeIII* (October 2011 vs. April 2011, August 2011 vs. February 2011, and August 2011 vs. May 2011) and none was significant for *MspI*. For both enzymes, less than 20% of the observed variation in bacterial community structure was explained by season and less than 36% was explained by month (in both nested and single factor analyses). The remaining variation was unexplained by these factors and may result from the colonization (or loss) of transient bacterial taxa unrelated to seasonal cycles and interspecific variation in the relative abundance of symbionts across host individuals. Statistical analyses of dispersion (PERMDISP) revealed no significant differences for both factors (season and months) and enzyme datasets (*HaeIII* and *MspI*; **Table 1**), indicating that heterogeneity within our data was not the main driver of structural differences retrieved for *D. fulgens* symbiont community. Bacterial communities associated with *D. fulgens* were significantly different from bacterioplankton communities (Supplementary Figure S2; PERMANOVA, *p* < 0.001 for both enzymes). Seawater bacterial communities exhibited spatially segregated clusters in composition except for December 2010 and March 2011, which overlapped to some degree (nMDS plot, Supplementary Figure S2). Overall, bacterioplankton communities showed clear seasonal shifts in composition, in contrast with our observations for ascidian-associated bacteria.

**Table 1:** Permutational statistical (PERMANOVA) and dispersion (PERMDISP) analyses of T-RFLP data to assess the bacterial community structure in *D. fulgens* over time.

		PERMANOVA		PERMDISP	
Factor	Enzyme	Pseudo-F^1^	P (perm)^2^	F	P (perm)^2^
Season	*HaeIII*	1.9406	0.009	1.5075	0.302
	*MspI*	1.9176	0.023	1.499	0.311
Month (within seasons)	*HaeIII*	1.8058	0.001	NA	NA
	*MspI*	1.6142	0.001	NA	NA
Month	*HaeIII*	2.171	0.001	2.071	0.433
	*MspI*	1.8612	0.001	4.1679	0.066

*Results are shown for the factors seasonal and month (within seasons), the single factor month, and for both restriction enzymes (*HaeIII* and *MspI*). NA: not analyzed.*

*^1^Pseudo-F = multivariate analog of Fisher’s *F* statistic (i.e., ratio of variance).*

*^2^P(perm) = Permutational P value (i.e., proportion of permuted pseudo-*F* statistics ≥ the original pseudo-F).*

Clone library analysis based on a near-full length fragment of the 16S rRNA gene resulted in 10 bacterial OTUs (99% sequence identity; **Table 2**) that represented a high coverage estimate (80.4%) of total diversity. All of the recovered sequences, except for two (OTUs 7 and 8), were affiliated to Proteobacteria and closely matched previously reported sequences isolated from either a sponge host or environmental/sediment samples (**Table 2**). In particular, most sequences (66.7%) corresponded to Alphaproteobacteria (OTUs 1, 2, 4, and 5), with the most common OTU (OTU 1, 38.1% of all sequences) matching closely (99%) to a sponge-associated bacterium collected near Monterey Harbor (CA, USA). OTUs 3 and 6 were affiliated to Gammaproteobacteria and matched most closely to bacteria described from environmental samples, specifically marine biofilms and seafloor lava, respectively. In contrast, the Gammaproteobacteria-affiliated OTU 9 was most similar (95.4% identity) to a bacterial sequence retrieved from the Mediterranean sponge *Tethya aurantium*. Finally, the singleton OTU 10 matched to a deltaproteobacterium sequence recovered from the Caribbean sponge *Plakortis* sp. (**Table 2**). Cyanobacteria (OTU 7) and Acidobacteria (OTU 8) sequences were also retrieved in our dataset. OTU 7 presented 97% sequence identity with coral-associated Cyanobacteria, while OTU 8 was identical to a sponge-associated bacterial sequence (**Table 2**).

**Table 2:** Bacterial OTUs obtained from clone library analyses, number of clones in each OTU, closest BLASTn match (identity percentage in parenthesis) and source, class and lowest taxonomic classification (confidence percentages in parenthesis).

OTU	Clones	T-RF abundance *HaeIII*/*MspI* (±SE)	BLASTn (acc. no., % match)	Phylum or class	Lowest taxon
1	8^a^	14.8/15.5 (±1.4/±1.7)	Sponge-associated (EU236387, 99)	Alphaproteobacteria (100)	G. *Hoeflea* (96)
2	3^a^	n.d./28.4^d^ (n.d./±1.9)	Sediment (GQ246350, 99.4)	Alphaproteobacteria (100)	G. *Rhodobium* (42)
3	2^b^	n.d./0.92 (n.d./±0.2)	Environmental (GQ274271, 98.1)	Gammaproteobacteria (100)	G. *Spongiibacter* (31)
4	2^b^	n.d./28.4^d^ (n.d./±1.9)	Sediment (FM242451, 97.3)	Alphaproteobacteria (100)	G. *Sneathiella* (100)
5	1^c^	7.7/28.4^d^ (±1.0/±1.9)	Environmental (HM591412, 99)	Alphaproteobacteria (100)	G. *Kordiimonas* (100)
6	1^b^	16.2/1.9 (±1.0/±1.2)	Environmental (EU491139, 93.4)	Gammaproteobacteria (100)	G. *Ectothiorhodosinus* (27)
7	1^b^	3.3/5.0 (±0.3/±0.8)	Coral-associated (GU119659, 97.4)	Cyanobacteria (97)	F. Chlorarachniophyceae (36)
8	1^b^	n.d./n.d. (n.d./n.d.)	Sponge-associated (FJ269336, 99.4)	Acidobacteria (99)	O. Gp21 (99)
9	1^b^	33.7/30.0 (±1.9/±1.9)	Sponge-associated (AM259846, 95.4)	Gammaproteobacteria (100)	G. *Ectothiorhodosinus* (42)
10	1^b^	n.d./n.d. (n.d./n.d.)	Sponge-associated (JX280383, 100)	Deltaproteobacteria (84)	G. *Desulfocurvus* (35)

*G., genus; F., family; and O., order. ^a^Present in both clone libraries. ^b^Present in DF2 library. ^c^Present in DF7 library. ^d^OTUs 2, 4, and 5 contribute to the same TRF (MspI digest).*

**FIGURE 2:**
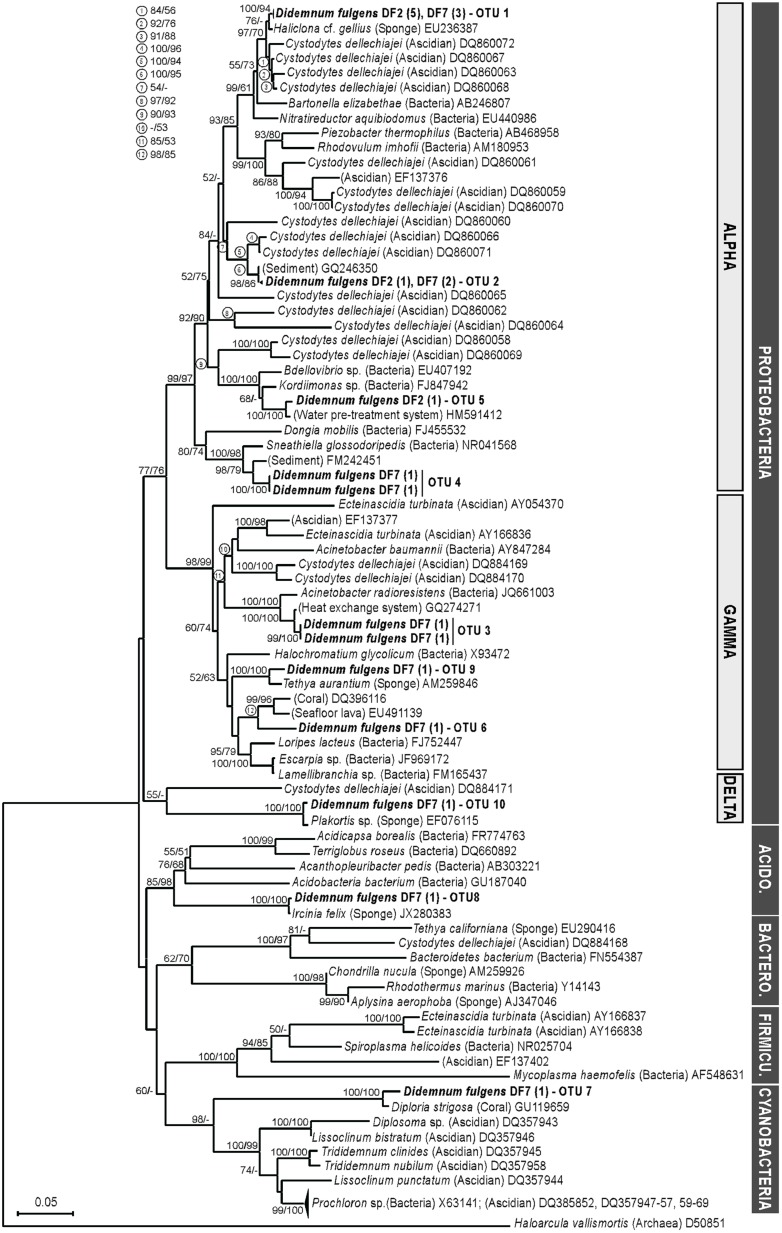
**Phylogeny of partial bacterial 16S rRNA gene sequences in *D. fulgens.*** Sequences obtained in this study are highlighted (bold lettering). Labels on terminal nodes of reference sequences indicate host or bacterial species, sources (in parenthesis) and GenBank accession numbers. Labels on terminal nodes of sequences from this study include sample name (DF2: April 2011 or DF7: May 2011), number of sequenced clones (in parenthesis) and ascribed OTU (as in **Table 2**). Tree topology was obtained from neighbor-joining (NJ) analysis. Individual bootstrap values from NJ and maximum likelihood (ML) analyses are located on the tree nodes or in the upper-left of the figure, corresponding to circle numbers on tree nodes. Dark gray bars indicate bacterial phylum: Proteobacteria, Acido.: Acidobacteria, Bactero.: Bacteroidetes, Firmicu.: Firmicutes, Cyanobacteria. Clear gray bars indicate bacterial class for the Proteobacteria. Scale bar represents 0.05 substitutions per site.

Phylogenetic analyses revealed that many of the sequences retrieved in this study formed well-supported clades (>90 bootstrap value) with bacterial sequences obtained from other ascidian species, in addition to the host-derived and environment sequences mentioned above (**Figure 2**). In particular, sequences forming OTUs 1 and 2 (the two most common clone library OTUs) clustered together with sequences retrieved from the Mediterranean colonial ascidian *Cystodytes dellechiajei*, also collected off the coast of Spain (**Figure 2**). Other well-supported clades (>95 bootstrap values) grouped sequences obtained in this study with either sponge-associated (OTUs 8, 9, and 10) or coral-associated bacteria (OTU 7), environmental samples (OTUs 3, 4, 5), or a mix thereof (OTU 6). Sequences belonging to the bacterial phyla Bacteroidetes and Firmicutes were also common in other ascidian species but were not observed in *D. fulgens* (**Figure 2**).

Comparisons of clone library and T-RFLP data matched most of the symbiont taxa identified through clone libraries (10 OTUs) with particular T-RFLP peaks (Supplementary Table S2). OTUs 2, 3, and 4 were not detected with the restriction enzyme *HaeIII* because the predicted peaks were out of range but were retrieved with the *MspI* enzyme. OTUs 2, 4, and 5 were detected with the restriction enzyme *MspI* and resulted in the same empirical T-RF (446 bp), thus all three OTUs contribute to the relative abundance of this peak (Supplementary Table S2). Neither OTU 8 nor 10 were detected in our T-RFLP profiles (Supplementary Table S2). While a higher diversity of bacterial taxa was recovered by T-RFLP (116 TRFs with *HaeIII*, 106 TRFs with *MspI*) compared to clone libraries (10 OTUs), the majority of these taxa (74–82%) were rare, transient peaks (occurring in less than 10% of samples). In contrast, the empirical T-RFs of the eight clone library OTUs were represented dominant symbiont taxa, accounting for 75.7% (*HaeIIII* data) and 81.72% (*MspI* data) of total profile peak areas, and were consistently retrieved over time (except for *MspI* peak 492 that was only retrieved in November and March 2011), further confirming the stability of these symbionts.

### Bacterial Distribution in the Tunic of Adults and Larvae

**FIGURE 3:**
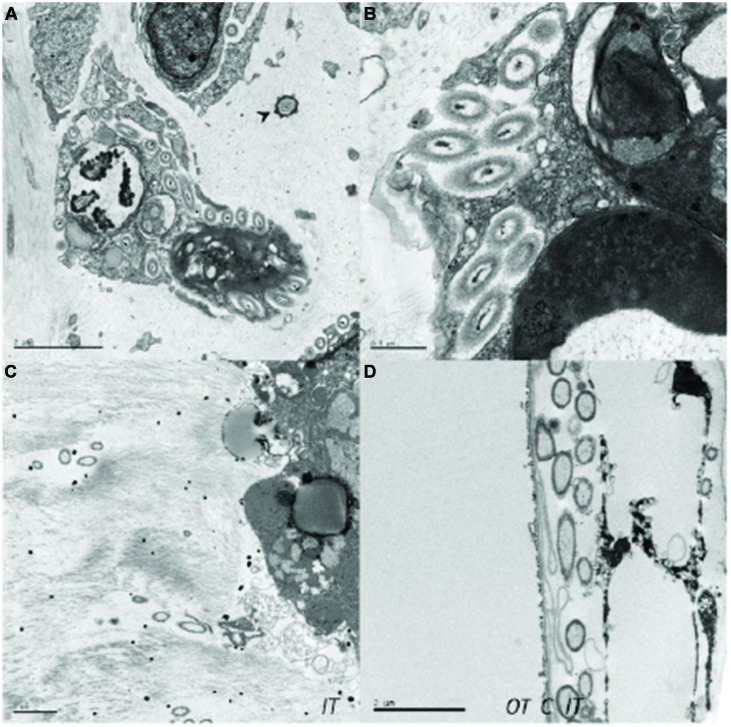
**Transmission electron microscopy images of bacterial cells observed in the adult tunic and in the larva of *D. fulgens.* (A)** Rod-shaped bacterial cells docked around ascidian cells and star-shaped bacterium (arrowhead); **(B)** detail of the bacterial docking; **(C)** bacterial distribution in the inner tunic of the larva, and **(D)** bacterial concentration at the larval cuticle edge (C, cuticle; IT, inner tunic; OT, outer tunic). Scale bars represent 2 μm **(A,C,D)** and 1 μm **(B)**.

Bacteria were abundant in the tunic of *D. fulgens*, especially surrounding the ascidian cells (**Figure 3A**). The main morphotype was a rod-shaped bacterium (up to 0.5 μm in diameter and 2 μm in length) that was generally found docked around animal cells and appeared to be actively phagocytosed (**Figure 3B**). Occasionally, other types of bacteria were observed, including a bacterium exhibiting a star-shaped appearance in transverse sections (**Figure 3A**). Rod-shaped bacteria were also found inside the tunic of a larva isolated from a mature *D. fulgens* colony. Contrary to observations of the adult tunic, the bacteria in the larva were not located around the animal cells but rather formed patches in the inner tunic (**Figure 3C**). A large number of bacteria were found in close association with the cuticle that separates the inner from the outer tunic of the larva (**Figure 3D**).

## Discussion

Monthly monitoring of *D. fulgens* colonies for over a year revealed high stability of ascidian-associated bacterial symbiont communities. Across all seasons and months, *D. fulgens* exhibited a bacterial symbiont community consisting mostly of Proteobacteria, in particular Alpha- and Gamma-proteobacteria. Clone library analysis resulted in 10 OTUs (99% sequence identity) that accounted for the majority of bacteria (relative abundance) represented in T-RFLP profiles. The empirical T-RFs assigned to each OTU were consistently retrieved over time, confirming the stability of these symbionts across seasonal environmental conditions (e.g., temperature fluctuations > 10°C). Other T-RFs were not consistently observed over time or among individual hosts and were considered as rare symbiont taxa. Variability in the rare microbiome occurred most prominently in warmer seasons (May to September), similarly to what was reported for sponges inhabiting the same region (Erwin et al., 2012b), and may coincide with thermal and food shortage stressors during these seasons. For example, a recent study of coral-associated bacteria suggested that microbial assemblages in corals inhabiting less preferred habitats exhibit higher diversity and less structure than corals located in more favorable habitats (Roder et al., 2015). Bacterial symbionts were abundant in the tunic of *D. fulgens* and were more commonly observed around animal cells in adult individuals and in the inner tunic of the larvae.

The NW Mediterranean Sea, including the Catalan coast of the Iberian Peninsula where this study was conducted, is characterized by clear seasonal trends in water temperature (e.g., López-Legentil et al., 2005b, 2013; Rius et al., 2009; Erwin et al., 2012b; Pineda et al., 2013), irradiance (e.g., Erwin et al., 2012b), and food availability (e.g., Coma et al., 2000). Annual temperature minima occur during the winter season and can fluctuate over 12°C at shallow sites (<7 m depth; Erwin et al., 2012b; López-Legentil et al., 2013). Irradiance conditions also exhibit a clear seasonal trend, with higher light intensity (2–3.5 times higher irradiance) and longer light duration periods in summer (up to 6 h more of light exposure per day) than in the winter (Erwin et al., 2012b). Food availability is generally low during summer because high temperature brings water stratification, which in turn results in a severe depletion of nutrients and suspended materials (Coma et al., 2000). These environmental factors, in particular temperature and food availability, have been shown to directly or indirectly influence the seasonality observed in the life cycle of many marine invertebrates (Becerro and Turon, 1992; Ribes et al., 1998; Coma et al., 2000, 2002; López-Legentil et al., 2005b, 2013). Moreover, resting stages are common in ascidians living in temperate seas and in the NW Mediterranean this phenomenon (called aestivation) typically occurs in summer (Turon, 1992; Turon and Becerro, 1992; Coma et al., 2000; López-Legentil et al., 2005b, 2013). Most *D. fulgens* colonies have been observed to enter a resting state after reproduction during the warmer months of the year (June to August; López-Legentil et al., 2013). However, none of these many possible factors (changing environmental conditions, different life cycle status of the animal) appeared to influence the dominant symbiotic bacterial communities inhabiting the ascidian tunic, a stark contrast to the seasonal shifts observed in free-living bacterioplankton communities (Schauer et al., 2003; Erwin et al., 2012b). Unique (Supplementary Figure S2) and stable (**Figure 1** and Supplementary Figure S1) bacterial communities in *D. fulgens* support the hypothesis of stable associations between bacteria and ascidian species and suggest that the ascidian tunic provides the symbionts with a unique and comparatively stable microbial habitat over time.

A lack of overall seasonal variation in symbiotic microbiota has also been reported in Mediterranean sponges (Thiel et al., 2007a,b, Erwin et al., 2012b), indicating that the temporal stability of bacterial symbiont communities may be widespread among Mediterranean invertebrates. Moreover, several of the OTUs retrieved in this study and in other ascidian-microbial symbiosis studies, closely matched sponge-associated bacterial symbionts (Martínez-García et al., 2007; Erwin et al., 2014). The existence of closely related bacterial symbionts within ascidians and sponges indicate that some bacterial lineages are adapted to host-associated lifestyles and can establish symbiotic associations with disparate host organisms. Since the dominant OTUs reported here were consistently retrieved across months and seasons, our data also suggest that these shared bacterial lineages among different taxa are able to maintain stable symbiotic relationships over time. Notably, the most dominant symbiont in *D. fulgens* was classified to the genus *Hoeflea*, a taxon that includes several symbiotic strains isolated from dinoflagellates (Biebl et al., 2006), cyanobacteria (Stevenson et al., 2011), and halophytes (Chung et al., 2013). In addition to the common trait of symbiotic association, *Hoeflea* species exhibit a size and morphology matching the dominant cell types visualized in *D. fulgens* adults and larvae (see below) and has a diverse metabolic repertoire, including photosynthesis (Biebl et al., 2006), iron-oxidation (Sorokina et al., 2012), and antibiotic (bacteriocin) production (Bentzon-Tilia et al., 2014). Additional studies targeting the function of ascidian-associated bacteria are required to further understand the metabolism of these symbionts and its potential benefits for the host.

Transmission electron microscopy observations revealed that most of the bacterial symbionts in the tunic of the didemnid *D. fulgens* were rod-shaped bacteria distributed around the animal cells. Occasionally, star-shaped bacteria were also observed in the tunic but were not associated with the animal cells. Similar symbiont morphologies and interactions with host cells have also been observed in the tunic of the Mediterranean colonial ascidian *C. dellechiajei* (F. Polycitoridae; Turon et al., 2005; Martínez-García et al., 2007). Thus, although most bacterial species are not identifiable by TEM, our observations suggest that rod- and star-shaped bacteria are common inhabitants in the ascidian tunic and that the animal is able to control bacterial population growth to some degree by phagocytosis. Clearly, further studies are needed to validate this hypothesis since few studies have paired microbial community descriptions in ascidians with TEM imaging.

Bacterial cells were also observed inside the inner tunic of *D. fulgens* larvae and in close association with the cuticle that separates the inner from the outer tunic of the larva. Within the larval tunic, bacterial cells were not located around the animal cells and thus none was observed being phagocytized. Rather, bacterial cells formed aggregates away from the animal cells, similar to what has been observed for the didemnid ascidian *Lissoclinum* aff. *fragile* (López-Legentil et al., 2011). The presence of bacterial cells in the larval tunic indicated that some of the bacterial symbionts described for the adult colonies are being vertically transmitted to progeny since horizontal transmission of these bacterial cells is unlikely (*D. fulgens* larva are brooded within the ascidian and not in direct exposure to ambient bacterioplankton). Vertical transmission of cyanobacterial photosymbionts has often been reported in ascidians and is assumed to be essential for host survival (reviewed in Hirose, 2014). However, no previous study has reported similar observations for bacterial symbionts other than Cyanobacteria. The TEM images obtained here suggest that, much like for the photosymbionts, other bacterial lineages are vertically transmitted to the ascidian progeny. Further studies utilizing taxa specific techniques such as fluorescence *in situ* hybridization (FISH) or next generation sequencing should cast some light on the identity of these symbionts. Combined with the stability exhibited by the *D. fulgens* microbiota, these results indicate that at least some bacterial symbionts may be indispensable for the establishment and long-term survival of ascidian colonies.

## Conflict of Interest Statement

The authors declare that the research was conducted in the absence of any commercial or financial relationships that could be construed as a potential conflict of interest.

## References

[B1] Aassila H. Bourguet-Kondracki M. L. Rifai S. Fassouane A. Guyot M. (2003). Idetification of Harman as the antibiotic compound produced by a tunicate-associated bacterium. *Marine Biotechnol.* 5 163–166. 10.1007/s10126-002-0060-7 12876652

[B2] Abdo Z. Schüette U. M. E. Bent S. J. Williams C. J. Forney L. J. Joyce P. (2006). Statistical methods for characterizing diversity of microbial communities by analysis of terminal restriction fragment length polymorphisms of 16S rRNA genes. *Environ. Microbiol.* 8 929–938. 10.1111/j.1462-2920.2005.00959.x 16623749

[B3] Anderson S. A. Northcote P. T. Page M. J. (2010). Spatial and temporal variability of the bacterial community in diferent chemotypes of the New Zealand marine sponge *Mycale* *hentscheli*. *FEMS Microbiol. Ecol.* 72 328–342. 10.1111/j.1574-6941.2010.00869.x 20412301

[B4] Balata D. Nesti U. Piazzi L. Cinelli F. (2007). Patterns of spatial variability of seagrass epiphytes in the north-west Mediterranean Sea. *Marine Biol.* 151 2025–2035. 10.1007/s00227-006-0559-y

[B5] Becerro M. A. Turon X. (1992). Reproductive cycles of the ascidians *Microcismus sabatieri* and *Halocynthia papillosa* in the Northwestern Mediterranean. *PSZN Marine Ecol.* 13 363–373. 10.1111/j.1439-0485.1992.tb00360.x

[B6] Behrendt L. Larkum A. W. D. Trampe E. Norman A. Sorensen S. J. Kühl M. (2012). Microbial diversity of biofilm communities in microniches associated with the didemnid ascidian *Lissoclinum patella*. *ISME J.* 6 1222–1237. 10.1038/ismej.2011.181 22134643 PMC3358027

[B7] Benjamini B. Y. Yekutieli D. (2001). The control of the false discovey rate in multiple testing under dependency. *Ann. Stat.* 29 1165–1188. 10.1186/1471-2105-9-114

[B8] Bentzon-Tilia M. Riemann L. Gram L. (2014). Draft genome sequence of *Hoeflea* sp. *strain BAL*378, a potential producer of bioactive compounds. *Genome Announc.* 2 e01213–e01214. 10.1128/genomeA.01213-14 25414510 PMC4239365

[B9] Biebl H. Tindall B. J. Pukall R. Lünsdorf H. Allgaier M. Wagner-Dobler I. (2006). *Hoeflea phototrophica* sp. nov., a novel marine aerobic alphaproteobacterium that forms bacteriochlorophyll a. *Int. J. Syst. Evol. Microbiol.* 56 821–826. 10.1099/ijs.0.63958-0 16585702

[B10] Blunt J. W. Copp B. R. Keyzers R. A. Munro M. H. G. Prinsep M. R. (2014). Marine natural products. *Nat. Prod. Rep.* 31 160–258. 10.1039/c3np70117d 24389707

[B11] Blunt J. W. Copp B. R. Keyzers R. A. Munro M. H. G. Prinsep M. R. (2015). Marine natural products. *Nat. Prod. Rep.* 32 116–211. 10.1039/c4np00144c 25620233

[B12] Bryon D. A. Scavia D. (2008). *An Integrated Assessment of the Continued Spread and Potential Impacts of the Colonial Ascidian, Didemnum sp. A, in U.S. Waters.* Silver Spring, MD: NOAA/National Centers for Coastal Ocean Science.

[B13] Chung E. J. Park J. A. Pramanik P. Bibi F. Jeon C. O. Chung Y. R. (2013). *Hoeflea suaedae* sp. nov., an endophytic bacterium isolated from the root of the halophyte *Suaeda maritima*. *Int. J. Syst. Evol. Microbiol.* 63 2277–2281. 10.1099/ijs.0.045484-0 23159752

[B14] Cole J. R. Chai B. Farris R. J. Wang Q. Kulam-Syed-Mohideen A. S. McGarrell D. M. (2007). The ribosomal database project (RDP-II): introducing myRDP space and quality controlled public data. *Nucleic Acids Res.* 35 D169–D172. 10.1093/nar/gkl889 17090583 PMC1669760

[B15] Coma R. Ribes M. Gili J.-M. Zabala M. (2000). Seasonality in coastal benthic ecosystems. *Trends Ecol. Evol.* 15 448–453. 10.1016/S0169-5347(00)01970-4 11050347

[B16] Coma R. Ribes M. Gili J.-M. Zabala M. (2002). Seasonality of in situ respiration rate in three temperate benthic suspension feeders. *Limnol. Oceanogr.* 47 324–331. 10.4319/lo.2002.47.1.0324

[B17] Culman S. W. Bukowski R. Gauch H. G. Cadillo-Quiroz H. Buckley D. H. (2009). T-REX: software for the processing and analysis of T-RFLP data. *BMC Bioinformatics* 10:171 10.1186/1471-2105-10-171 PMC2702334 19500385

[B18] Erwin P. M. López-Legentil S. Gonzalez-Pech R. Turon X. (2012a). A specific mix of generalists: bacterial symbionts in Mediterranean *Ircinia* spp. *FEMS Microbiol. Ecol.* 79 619–637. 10.1111/j.1574-6941.2011.01243.x 22092516

[B19] Erwin P. M. Pita L. López-Legentil S. Turon X. (2012b). Stability of sponge-associated bacteria over large seasonal shifts in temperature and irradiance. *Appl. Environ. Microbiol.* 78 7358–7360. 10.1128/AEM.02035-12 22885741 PMC3457113

[B20] Erwin P. M. Pineda M. C. Webster N. Turon X. López-Legentil S. (2013). Small core communities and high variability in bacteria associated with the introduced ascidian *Styela plicata*. *Symbiosis* 59 35–46. 10.1007/s13199-012-0204-0

[B21] Erwin P. M. Pineda M. C. Webster N. S. Turon X. López-Legentil S. (2014). Down under the tunic: bacterial biodiversity hotspots and widespread ammonia-oxidizing archaea in coral reef ascidians. *ISME J.* 8 575–588. 10.1038/ismej.2013.188 24152714 PMC3930322

[B22] Erwin P. M. Thacker R. W. (2007). Phylogenetic analysis of marine sponges within the order Verongida: a comparsion of morphological and molecular data. *Invertebr. Biol.* 126 220–234. 10.1111/j.1744-7410.2007.00092.x

[B23] Felsenstein J. (1985). Confidence limits on phylogenies: an approach using the bootstrap. *Evolution* 39 783–791. 10.2307/2408678 28561359

[B24] Hernández-Mariné M. Turon X. Catalan J. (1990). A marine *Synechocystis* (Chroococcales, Cyanophyta) epizoic on didemnid ascidians from the Mediterranean Sea. *Phycologia* 29 275–284.

[B25] Hirose E. (2000). Plant rake and algal pouch of the larvae in the tropical ascidian *Diplosoma similis*: an adaptation for vertical transmission of photosynthetic symbionts *Prochloron* sp. *Zool. Sci.* 17 233–240. 10.2108/zsj.17.233

[B26] Hirose E. (2013). Didemnid ascidians harboring cyanobacteria from Kumejima Island and Tonakijima Island, Ryukyu Archipelago, Japan. *Biol. Mag. Okinawa* 51 41–49.

[B27] Hirose E. (2014). Ascidian photosymbiosis: diversity of cyanobacterial transmission during embryogenesis. *Genesis* 53 121–131. 10.1002/dvg.22778 24700539

[B28] Hirose E. Akahori M. Oka A. T. Kurabayashi A. (2004). Some *Prochloron*-bearing didemnid ascidians collected from the reef shores of Iriomote Island (Okinawa, Japan). *Biol. Mag. Okinawa* 42 7–15.

[B29] Hirose E. Fukuda T. (2006). Vertical transmission of photosymbionts in the colonial ascidian *Didemnum molle*: the larval tunic prevents symbionts from attaching to the anterior part of larvae. *Zool. Sci.* 23 669–674. 10.2108/zsj.23.669 16971784

[B30] Hirose E. Kamijoh A. Oka A. T. (2007). Distribution of the photosymbiotic ascidians in Chichijima Island (Ogasawara Islands. Tokyo). *Biol. Mag. Okinawa* 45 3–9.

[B31] Hirose E. Maruyama T. Cheng L. Lewin R. A. (1996). Intracellular symbiosis of a photosynthetic prokaryote, *Prochloron* sp., in a colonial ascidian. *Invertebr. Biol.* 115 343–348. 10.2307/3227023

[B32] Hirose E. Nozawa Y. (2010). Photosymbiotic ascidians from Kenting and Lyudao in Taiwan. *Zool. Stud.* 49 681–687. 10.6620/ZS.2020.59-19 PMC7688397 33262843

[B33] Hirose E. Oka A. T. Akahori M. (2005). Sexual reproduction of the photosymbiotic ascidian *Diplosoma virens* in the Ryukyu Archipelago, Japan: vertical transmission, seasonal change, and possible impact of parasitic copepods. *Marine Biol.* 146 677–682.

[B34] Kearse M. Wilson M. R. Stones-Havas S. Cheung M. Sturrock S. Buxton S. (2012). Geneious Basic: an integrated and extendable desktop software platform for the organization and analysis of sequence data. *Bioinformatics* 28 1647–1649. 10.1093/bioinformatics/bts199 22543367 PMC3371832

[B35] Kojima A. Hirose E. (2010). Transfer of prokaryotic algal symbionts from a tropical ascidian (*Lissoclinum punctatum*) colony to its larvae. *Zool. Sci.* 27 124–127. 10.2108/zsj.27.124 20235396

[B36] Kojima A. Hirose E. (2012). Transmission of cyanobacterial symbionts during embryogenesis in the coral eeef ascidians *Trididemnum nubilum* and *T*. *clinides* (Didemnidae. Ascidiacea, Chordata). *Biol. Bull.* 222 63–73. 22426633 10.1086/BBLv222n1p63

[B37] Koukouras A. Voultsiadou-Koukouras E. Kebrekidis T. Vafifis D. (1995). Ascidian fauna of the Aegean Sea with a check list of the Eastern Mediterranean and Black Sea species. *Ann. l’Inst. Océanogr.* 71 19–34.

[B38] Lafargue F. Duclaux G. (1979). Premier example, en Atlantique tropical, d’une association symbiotique entre une ascidie Didemnidae et une cyanophycée Chroococcale: *Trididemnum cyanophorum* nov. sp. et *Synechocystis trididemni* nov. sp. *Ann. l’Inst. Océanogr.* 55 163–184.

[B39] Lafargue F. Wahl M. (1987). The didemnid ascidian fauna of France. *Ann. l’Inst. Océanogr.* 63 1–46.

[B40] Lambert C. C. (2005a). Historical introduction, overview, and reproductive biology of the protochordates. *Can. J. Zool.* 83 1–7. 10.1139/z04-160

[B41] Lambert G. (2005b). Ecology and natural history of the protochordates. *Can. J. Zool.* 83 34–50. 10.1139/z04-156

[B42] Lambert G. (2007). Invasive sea squirts: a growing global problem. *J. Exp. Marine Biol. Ecol.* 342 3–4. 10.1016/j.jembe.2006.10.009

[B43] Larkin M. A. Blackshields G. Brown N. P. Chenna R. McGettigan P. A. McWilliam H. (2007). ClustalW and ClustalX version 2. *Bioinformatics* 23 2947–2948. 10.1093/bioinformatics/btm404 17846036

[B44] Lewin R. A. (1977). *Prochloron*, type genus of the Prochlorophyta. *Phycologia* 16:217 10.2216/i0031-8884-16-2-217.1

[B45] Lewin R. A. (1978). “Distribution of symbiotic didemnids associated with prochlorophytes,” *in Proceedings of the International Symposium of Marine Biogeography and Evolution in the Southern Hemisphere* (Auckland: New Zealand DSIR Information Series), 137 365–369.

[B46] López-Legentil S. Bontemps-Subielos N. Turon X. Banaigs B. (2006). Temporal variation in the production of four secondary metabolites in a colonial ascidian. *J. Chem. Ecol.* 32 2079–2084. 10.1007/s10886-006-9148-2 16924427

[B47] López-Legentil S. Bontemps-Subielos N. Turon X. Banaigs B. (2007). Secondary metabolite and inorganic contents in *Cystodytes* sp. (Ascidiacea): temporal patterns and association with reproduction and growth. *Marine Biol.* 151 293–299. 10.1007/s00227-006-0472-4

[B48] López-Legentil S. Dieckmann R. Bontemps-Subielos N. Turon X. Banaigs B. (2005a). Qualitative variation of alkaloids in color morphs of *Cystodytes* (Ascidiacea). *Biochem. Syst. Ecol.* 33 1107–1119. 10.1016/j.bse.2005.03.011

[B49] López-Legentil S. Ruchty M. Domenech A. Turon X. (2005b). Life cycles and growth rates of two morphotypes of *Cystodytes* (Ascidiacea) in the western Mediterranean. *Marine Ecol. Prog. Ser.* 296 219–228. 10.3354/meps296219

[B50] López-Legentil S. Erwin P. M. Velasco M. Turon X. (2013). Growing or reproducing in a temperate sea: optimization of resource allocation in a colonial ascidian. *Invertebr. Biol.* 132 69–80. 10.1111/ivb.12013

[B51] López-Legentil S. Song B. Bosch M. Pawlik J. R. Turon X. (2011). Cyanobacterial diversity and a new *Acaryochloris*-like symbiont from Bahamian sea-squirts. *PLoS ONE* 6:e23938 10.1371/journal.pone.0023938 PMC3161822 21915246

[B52] Martínez-García M. Díaz-Valdés M. Wanner G. Ramos-Esplà A. Antón J. (2007). Microbial community associated with the colonial ascidian *Cystodytes* *dellechiajei*. *Environ. Microbiol.* 9 521–534. 10.1111/j.1462-2920.2006.01170.x 17222150

[B53] Martínez-García M. Koblízek M. López-Legentil S. Antón J. (2011). Epibiosis of oxygenic phototrophs containing chlorophylls a, b, c, and d on the colonial ascidian *Cystodytes* *dellechiajei. Microb. Ecol.* 61 13–19. 10.1007/s00248-010-9694-6 20532497

[B54] Martinez-Murcia A. J. Acinas S. G. Rodriguez-Valera F. (1995). Evaluation of prokaryotic diversity by restrictase digestion of 16S rDNA directly amplified from hypersaline environments. *FEMS Microbiol. Ecol.* 17 247–255. 10.1016/0168-6496(95)00029-A

[B55] Millar R. H. (1971). The biology of ascidians. *Adv. Marine Biol.* 9 1–100. 10.1016/S0065-2881(08)60341-7

[B56] Milne-Edwards H. (1841). Observations sur les ascidies composées des côtes de La Manche. *Mem. Acad. Sci. Inst. Fr.* 18 217–326.

[B57] Miyashita H. Ikemoto H. Kurano N. Adachi K. Chihara M. Miyachi S. (1996). Chlorophyll d as a major pigmant. *Nature* 383:402 10.1038/383402a0

[B58] Miyashita H. Ikemoto H. Kurano N. Miyachi S. Chihara M. (2003). *Acaryochloris marina* gen. et sp. nov. (cyanobacteria), an oxygenic photosynthetic prokaryote containing chl d as a major pigment. *J. Phycol.* 39 1247–1253. 10.1111/j.0022-3646.2003.03-158.x

[B59] Münchhoff J. Hirose E. Maruyama T. Sunairi M. Burns B. P. Neilan B. A. (2007). Host specificity and phylogeography of the prochlorophyte *Prochloron* sp., an obligate symbiont in didemnid ascidians. *Environ. Microbiol.* 9 890–899. 10.1111/j.1462-2920.2006.01209.x 17359261

[B60] Oka A. T. Hirose E. (2008). Photosymbiotic ascidians from Nakanoshima Island and Takarajima Island (the Tokara Islands, Ryukyu Archipelago, Japan) with remarks on the status of *Siplosoma midori* (Tokioka, 1954). *Publications Seto Marine Biol. Lab.* 40 85–92.

[B61] Paul V. J. Ritson-Williams R. Sharp K. (2011). Marine chemical ecology in benthic environments. *Nat. Prod. Rev.* 28 345–387. 10.1039/C0NP00040J 21125086

[B62] Pineda M. C. López-Legentil S. Turon X. (2013). Year-round reproduction in a seasonal sea: biological cycle of the introduced ascidian *Styela plicata* in the Western Mediterranean. *Marine Biol.* 160 221–230. 10.1007/s00227-012-2082-7

[B63] Pita L. Turon X. López-Legentil S. Erwin P. M. (2013). Host rules: spatial stability of bacterial communities with marine sponges (*Ircinia* spp.) in the Western Mediterranean Sea. *FEMS Microbiol. Ecol.* 86 268–276. 10.1111/1574-6941.12159 23837533

[B64] Reynolds E. S. (1963). The use of lead citrate at high pH as an electron-opaque stain in electron microscopy. *J. Cell Biol.* 17 208–212. 10.1083/jcb.17.1.208 13986422 PMC2106263

[B65] Ribes M. Coma R. Gili J. (1998). Seasonal variation of in situ feeding rates by the temperate ascidian *Halocynthia papillosa*. *Marine Ecol. Prog. Ser.* 175 201–213. 10.3354/meps175201

[B66] Riesenfeld C. S. Murray A. E. Baker B. J. (2008). Characterization of the microbial community and polyketide biosynthetic potential in the palmerolide-producing tunicate *Synoicum adareanum*. *J. Nat. Prod.* 71 1812–1818. 10.1021/np800287n 18950228

[B67] Ritzmann N. F. da Rocha R. M. Roper J. J. (2009). Sexual and asexual reproduction in *Didemnum rodriguesi* (Ascidiacea. Didemnidae). *Iheringia Sér. Zool.* 99 106–110. 10.1590/S0073-47212009000100015

[B68] Rius M. Pineda M. C. Turon X. (2009). Population dynamics and life cycle of the introduced ascidian *Microcosmus squamiger* in the Mediterranean Sea. *Biol. Invasions* 11 2181–2194. 10.1007/s10530-008-9375-2

[B69] Roder C. Bayer T. Aranda M. Kruse M. Voolstra C. R. (2015). Microbiome structure of the fungid coral *Ctenactis echinata* align with environmental differences. *Mol. Ecol.* 24 3501–3511. 10.1111/mec.13251 26018191 PMC4736464

[B70] Schauer M. Balague V. Pedros-Alio C. Massana R. (2003). Seasonal changes in the taxonomic composition of bacterioplankton in a coastal oligitrophic system. *Aquat. Microb. Ecol.* 3 163–174. 10.3354/ame031163

[B71] Schmidt E. W. Donia M. S. (2010). Life in cellulose houses: symbiotic bacterial biosynthesis of ascidian drugs and drug leads. *Curr. Opin. Biotechnol.* 21 827–833. 10.1016/j.copbio.2010.10.006 21050742 PMC2992989

[B72] Schmidt E. W. Nelson J. T. Rasko D. A. Sudek S. Eisen J. A. Haygood M. G. (2005). Patellamide A and C biosynthesis by a microcin-like pathway in *Prochloron didemni*, the cyanobacterial symbiont of *Lissoclinum patella*. *Proc. Natl. Acad. Sci. U.S.A.* 102 7315–7320. 10.1073/pnas.0501424102 15883371 PMC1091749

[B73] Schuett C. Doepke H. Groepler W. Wichels A. (2005). Diversity of intratunical bacteria in the tunic matrix of the colonial ascidian *Diplosoma migrans*. *Helgoland Marine Res.* 59 136–140. 10.1007/s10152-004-0212-4

[B74] Shenkar N. Loya Y. (2008). The solitary ascidian Herdmania momus: native (Red Sea) versus non-indigenous (Mediterranean) populations. *Biol. Invasions* 10 1431–1439. 10.1007/s10530-008-9217-2

[B75] Shenkar N. Swalla B. J. (2011). Global diversity of Ascidiacea. *PLoS ONE* 6:e20657 10.1371/journal.pone.0020657 PMC3119061 21701684

[B76] Shimada A. Yano N. Kanai S. Lewin R. A. Maruyama T. (2003). Molecular phylogenetic relationship between two symbiotic photo-oxygenic prokaryotes. *Prochloron* sp. and *Synechocystis trididemni. Phycologia* 42 193–197.

[B77] Smith E. P. van Belle G. (1984). Nonparametric estimation of species richness. *Biometrics* 40 119–129. 10.2307/2530750

[B78] Sorokina A. Y. Chernousova E. Y. Dubinina G. A. (2012). *Hoeflea siderophila* sp. nov., a new neutrophilic iron-oxidizing bacterium. *Microbiology* 81 59–66. 10.1134/S0026261712010146 22629683

[B79] Stevenson B. S. Suflita M. T. Stamps B. W. Moore E. R. Johnson C. N. Lawson P. A. (2011). *Hoeflea anabaenae* sp. nov., an epiphytic symbiont that attaches to the heterocysts of a strain of *Anabaena*. *Int. J. Syst. Evol. Microbiol.* 61 2439–2444. 10.1099/ijs.0.025353-0 21075905

[B80] Tait E. Carman M. Sievert S. M. (2007). Phylogenetic diversity of bacteria associated with ascidians in Eel Pond (Woods Hole, Massachusetts, USA). *J. Exp. Marine Biol. Ecol.* 342 138–146. 10.1016/j.jembe.2006.10.024

[B81] Tamura K. Dudley J. Nei M. Kumar S. (2007). MEGA4: molecular evolutionary genetics analysis (MEGA) software version 4.0. *Mol. Biol. Evol.* 24 1596–1599. 10.1093/molbev/msm092 17488738

[B82] Tavaré S. (1986). “Some probabilistic and statistical problems in the analysis of DNA sequences,” in *Some Mathematical Questions in Biology – DNA Sequence Analysis* ed. Miura R. M. (Providence, RI: American Mathematics Society) 57–86.

[B83] Thiel V. Leininger S. Schmaljohann R. Brümmer F. Imhoff J. (2007a). Sponge-specific bacterial associations of the mediterranean sponge *Chondrilla nucula* (Demospongiae. Tetractinomorpha). *Microb. Ecol.* 54 101–111. 10.1007/s00248-006-9177-y 17364249

[B84] Thiel V. Neulinger S. C. Staufenberger T. Schmaljohann R. Imhoff J. F. (2007b). Spatial distribution of sponge - associated bacteria in the Mediterranean sponge *Tethya aurantium*. *FEMS Microbiol. Ecol.* 59 47–63. 10.1111/j.1574-6941.2006.00217.x 17059482

[B85] Tianero M. D. B. Kwan J. C. Wyche T. P. Presson A. P. Koch M. Barrows L. R. (2015). Species specificity of symbiosis and secondary metabolism in ascidians. *ISME J.* 9 615–628. 10.1038/ismej.2014.152 25171330 PMC4331574

[B86] Turner S. Pryer K. M. Miao V. P. W. Palmer J. D. (1999). Investigating deep phylogenetic relationships among cyanobacteria and plastids by small subunit rRNA sequence analysis. *J. Eukaryot. Microbiol.* 46 327–338. 10.1111/j.1550-7408.1999.tb04612.x 10461381

[B87] Turon X. (1992). Periods of non-feeding in Polysyncraton lacazei (Ascidiacea: Didemnidae): a rejuvenative process? *Marine Biol.* 112 647–655. 10.1007/BF00346183

[B88] Turon X. Becerro M. A. (1992). Growth and survival of several ascidian species from the northwestern Mediterranean. *Marine Ecol. Prog. Ser.* 82 235–247. 10.3354/meps082235

[B89] Turon X. López-Legentil S. Banaigs B. (2005). Cell types, microsymbionts, and pyridoacridine distribution in the tunic of three color morphs of the genus *Cystodytes* (Ascidiacea, Polycitoridae). *Invertebr. Biol.* 124 355–369. 10.1111/j.1744-7410.2005.00033.x

[B90] Valentine P. C. Carman M. R. Blackwood D. S. Heffron E. J. (2007). Ecological observations on the colonial ascidian *Didemnum* sp. in a New England tide pool habitat. *J. Exp. Marine Biol. Ecol.* 342 109–121. 10.1016/j.jembe.2006.10.021

[B91] Velasco M. (2012). *Reproductive Cycle and Growth Rate in the Colonial Ascidian Didemnum fulgens.* MS thesis, Master in Biodiversity, University of Barcelona, Barcelona.

[B92] Yokobori S. Kurabayashi A. Neilan B. A. Maruyama T. Hirose E. (2006). Multiple origins of the ascidian-*Prochloron* symbiosis: molecular phylogeny of photosymbiotic and non-symbiotic colonial ascidians inferred from 18S rDNA sequences. *Mol. Phylogenet. Evol.* 40 8–19. 10.1016/j.ympev.2005.11.025 16531073

[B93] York A. Gallager S. Taylor R. Vine N. Lerner S. (2008). Using a towed optical habitat mapping system to monitor the invasive tunicate species *Didemnum* sp. along the norheast continental shelf. *Oceans* 2008 1–9.

