# Supplementary material for: Temporal stability of bacterial symbionts in a temperate ascidian
Source: Front Microbiol. 2015 Sep 24;6:1022. doi: 10.3389/fmicb.2015.01022 (PMC4585324; doi:10.3389/fmicb.2015.01022)
Supplement: Supplementary file 1 [file Table_1.DOCX]

**Supplementary material**

**Table S1.** Sampling dates and replicate numbers for T-RFLP profiling.

|  | **Dataset (Restriction Enzyme)** | |
| --- | --- | --- |
| **Sampling date** | *Hae III* | *Msp I* |
| 26 August 2010 | 3 | 2 |
| 16 February 2011 | 4 | 4 |
| 31 March 2011 | 4 | 4 |
| 28 April 2011 | 5 | 4 |
| 26 May 2011 | 4 | 4 |
| 17 June 2011 | 3 | 3 |
| 21 July 2011 | 4 | 4 |
| 11 August 2011 | 5 | 5 |
| 9 September 2011 | 4 | 2 |
| 12 October 2011 | 5 | 5 |
| 10 November 2011 | 3 | 3 |
| 10 December 2011 | 2 | 2 |
| 12 January 2012 | 3 | 3 |
| 16 March 2012 | 4 | 2 |
| 12 April 2012 | 4 | 3 |
| 18 May 2012 | 3 | 3 |
| ***Total =*** | **60** | **53** |
| ***Average (per month) =*** | **3.75** | **3.31** |
