# Supplementary material for: Temporal stability of bacterial symbionts in a temperate ascidian
Source: Front Microbiol. 2015 Sep 24;6:1022. doi: 10.3389/fmicb.2015.01022 (PMC4585324; doi:10.3389/fmicb.2015.01022)
Supplement: Supplementary file 2 [file Table_2.DOCX]

**Supplementary material**

**Table S2.** Matching of clone library sequences and T-RFLP profile peaks after *in silico* digestion. n.d. = not detected, * = Predicted peak out of range

|  | **HaeIII** | | |  | **MspI** | | |
| --- | --- | --- | --- | --- | --- | --- | --- |
| **OTU** | Predicted Peak (bp) | Empirical Peak (bp) | Relative Abund ±SE |  | Predicted Peak (bp) | Empirical Peak (bp) | Relative Abund ±SE |
| OTU 1 | 220 | 222 | 14.8 ±1.4 |  | 434 | 433 | 15.5 ±1.7 |
| OTU 2 | 33 | * | * |  | 448 | 446* | 28.4 ±1.9 |
| OTU 3 | 33 | * | * |  | 484 | 483 | 0.92 ±0.2 |
| OTU 4 | 33 | * | * |  | 446 | 446* | 28.4 ±1.9 |
| OTU 5 | 199 | 199 | 7.7 ±1.0 |  | 447 | 446* | 28.4 ±1.9 |
| OTU 6 | 322 | 322 | 16.2 ±1.0 |  | 494 | 492 | 1.9 ±1.2 |
| OTU 7 | 304 | 302 | 3.3 ± 0.3 |  | 506 | 507 | 5.0 ±0.8 |
| OTU 8 | 167 | n.d. | --- |  | 274 | n.d. | --- |
| OTU 9 | 255 | 256 | 33.7 ±1.9 |  | 493 | 491 | 30.0 ±1.9 |
| OTU 10 | 278 | n.d. | --- |  | 157 | n.d. | --- |

*Note that OTUs 2, 4 and 5 result in the same empirical T-RFs.
