# Supplementary material for: Temporal stability of bacterial symbionts in a temperate ascidian
Source: Front Microbiol. 2015 Sep 24;6:1022. doi: 10.3389/fmicb.2015.01022 (PMC4585324; doi:10.3389/fmicb.2015.01022)

Supplementary material

Figure S1. Non-metric multi-dimensional scaling (nMDS) plots of bacterial community similarity in *Didemnum fulgens* over the 16 months of study. nMDS ordination based on Bray-Curtis similarity of T-RFLP profiles for *HaeIII* (A) and *MspI* (B) datasets. Stress values for two-dimensional ordination are shown in parenthesis for each enzyme.

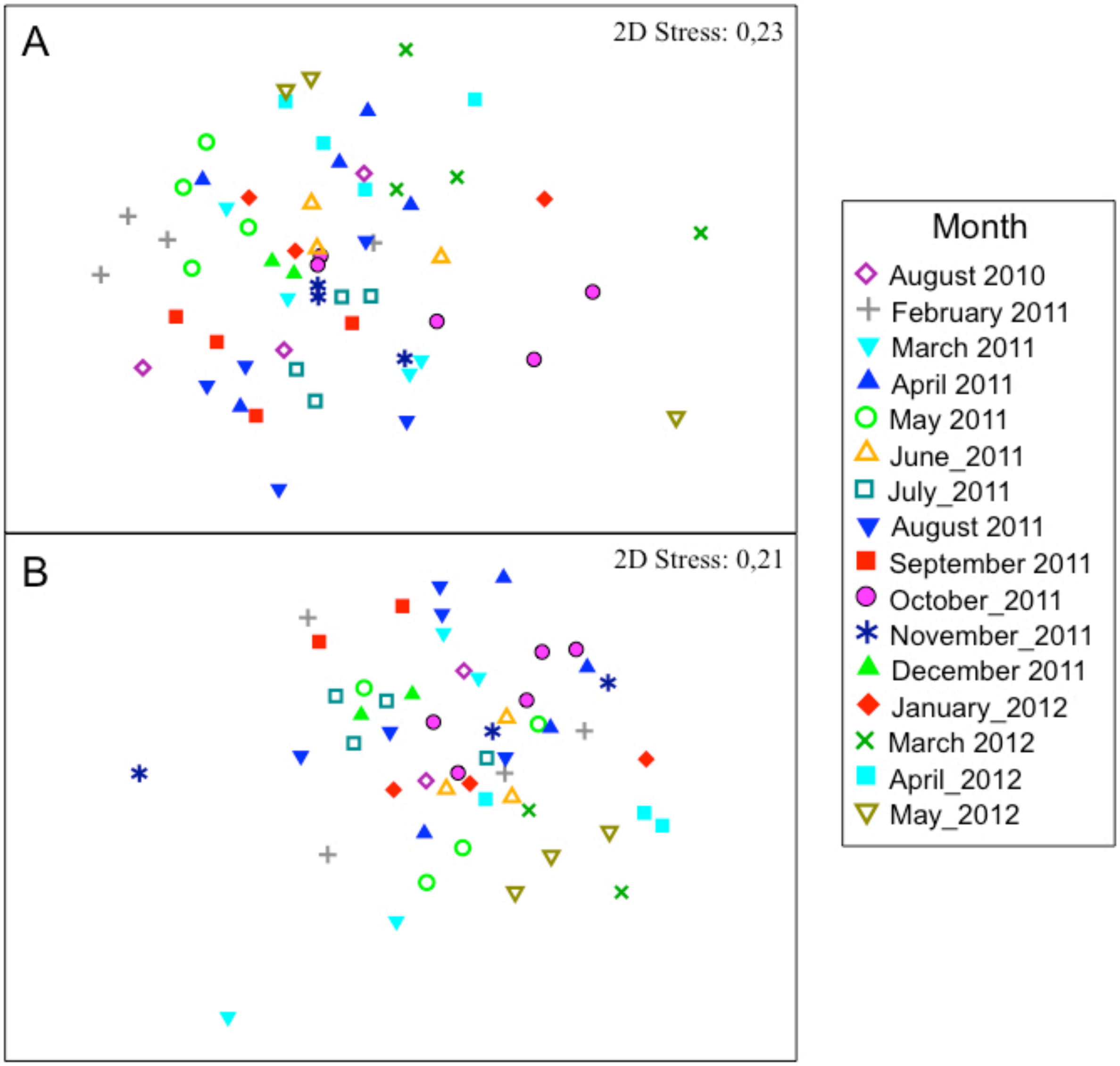

Supplement: Supplementary file 3 [file Image_1.PDF]
