# Supplementary material for: Temporal stability of bacterial symbionts in a temperate ascidian
Source: Front Microbiol. 2015 Sep 24;6:1022. doi: 10.3389/fmicb.2015.01022 (PMC4585324; doi:10.3389/fmicb.2015.01022)

Supplementary material

Figure S2. Nonmetric multidimensional scaling (nMDS) plots of bacterial community structure from *Didemnum fulgens* and ambient seawater. nMDS ordination based on Bray-Curtis similarity of T-RFLP profiles for (A) *HaeIII*, and (B) *MspI* data sets. Stress values for two-dimensional ordination are shown in the upper right corner of the graph. Continuous circles encompass data for *D. fulgens*, dashed circles for seawater samples. PERMANOVA p (perm) = 0.001 for both restriction enzymes; PERMDISP p (perm) = 0.096 for *HaeIII* and p (perm) = 0.001 for *MspI*.

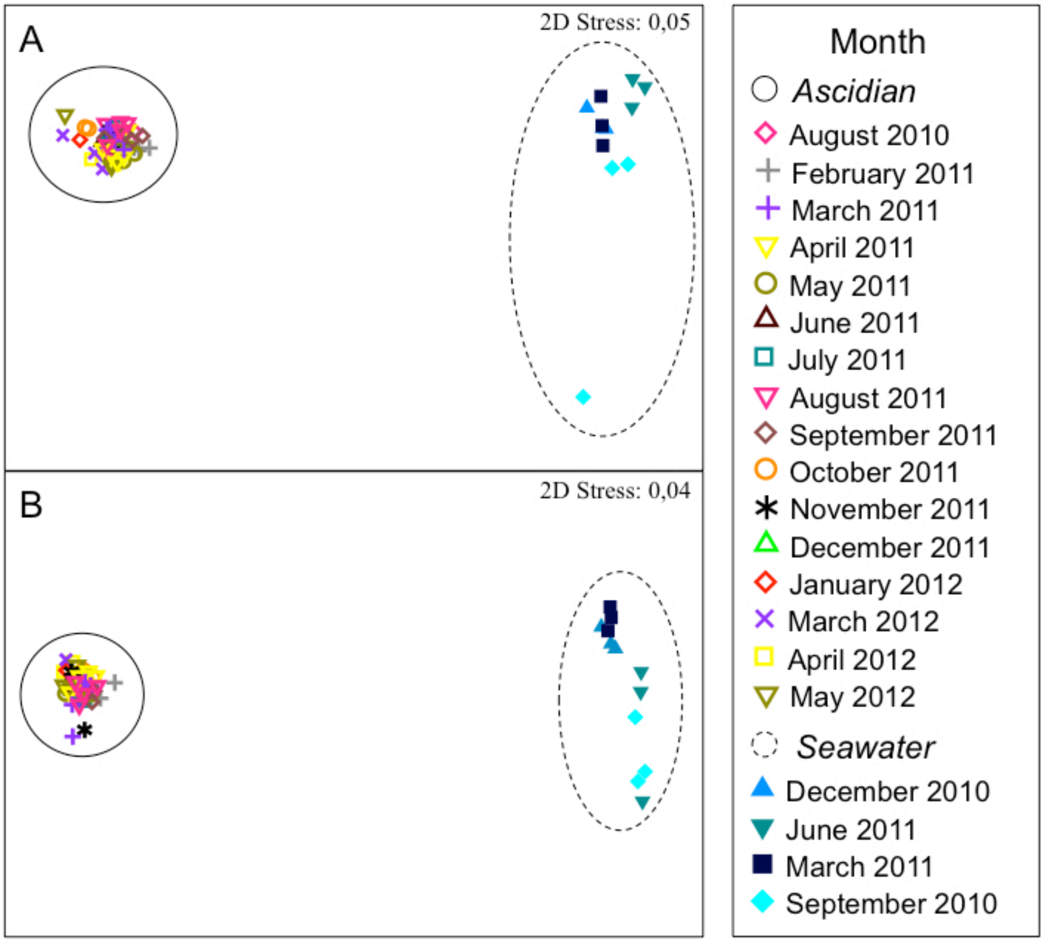

Supplement: Supplementary file 4 [file Image_2.PDF]
